# Explainable Machine Learning for Multicomponent Concrete: Predictive Modeling and Feature Interaction Insights

**DOI:** 10.3390/ma18194456

**Published:** 2025-09-24

**Authors:** Jie Wang, Junqi Deng, Siyi Li, Weijie Du, Zengqi Zhang, Xiaoming Liu

**Affiliations:** 1 School of Metallurgical and Ecological Engineering, University of Science and Technology Beijing, Beijing 100083, China; d202210142@xs.ustb.edu.cn (J.W.);; 2 State Key Laboratory of Advanced Metallurgy, University of Science and Technology Beijing, Beijing 100083, China

**Keywords:** multicomponent concrete, strength, machine learning, interaction

## Abstract

Multicomponent concrete is a widely used industrial material, yet its performance evaluation still relies heavily on expert judgment and long-term monitoring. With the rapid development of artificial intelligence (AI), machine learning has emerged as a promising tool in building science for analyzing complex datasets and reducing uncertainties associated with human factors. This study applies a variety of machine learning techniques—including linear and polynomial regressions, tree-based algorithms (Decision Tree, Random Forest, ExtraTrees, AdaBoost, CatBoost, and XGBoost), and the TabPFN model—to investigate the key factors influencing concrete compressive strength. To enhance interpretability, SHAP analysis was employed to uncover feature importance and interactions, offering new insights into the underlying mechanisms of multicomponent concrete. The findings provide a data-driven approach to support engineering design, facilitate decision-making in construction practice, and contribute to the development of more efficient and sustainable building materials.

## 1. Introduction

As urbanization continues, the global population has exceeded 7 billion, with approximately half residing in urban areas [1]. Cement concrete, the most widely produced industrial material by volume, is extensively used in the construction of housing, roads, bridges, dams, and other types of infrastructure [2]. The increasing demand for concrete has driven the widespread use of supplementary materials, such as ground granulated blast furnace slag and pulverized coal fly ash, in concrete production over the past two decades [3]. The incorporation of these diverse admixtures and recycled aggregates has improved material utilization but has also complicated conventional concrete mix designs. As a result, traditional proportioning theories encounter growing limitations in both research and practice, as they struggle to fully account for the complex interactions among multiple raw materials and additives. Consequently, the design and engineering implementation of multicomponent concrete mixtures has become a critical challenge, requiring new approaches and advanced tools for accurate performance evaluation and optimization.

Compressive strength, a primary indicator of concrete performance and an essential parameter in structural design, results from the complex interplay of multiple variables. These variables interact in nonlinear ways, making strength prediction challenging. Although conventional laboratory testing remains a reliable method for assessing compressive strength, it often requires significant time and cost, which limits its efficiency in practical engineering applications.

Traditional investigations have explored the influence of macroscopic parameters, such as curing conditions, raw material content, admixture dosage, and water-cement ratio [4], as well as microscopic characteristics, such as morphology [5], elemental migration, and phase transformation [6]. However, these approaches often treat variables in isolation, paying insufficient attention to the intrinsic properties of raw materials and the interdependence among influencing factors. As a result, findings across studies are frequently inconsistent or even contradictory.

To address these challenges, researchers have proposed various prediction strategies based on statistical analysis [7], empirical models [8] and numerical simulations [9]. Although these methods provide valuable insights, they are often time-consuming and may struggle to fully capture the nonlinear nature of strength development in concrete. With the continued advancement of artificial intelligence and machine learning, data-driven models have emerged as promising alternatives [10]. By learning from large volumes of mixed proportion data and corresponding experimental results, these models are capable of identifying complex relationships among variables and improving the accuracy and efficiency of compressive strength prediction.

In recent years, various machine learning algorithms applied in the field of materials science have made significant progress [11]. Decision trees and ensemble methods have shown potential for predicting the strength of multicomponent concrete and may gradually replace empirical formulas and traditional theoretical calculations in this area. TabPFN (Tabular Prior-data Fitted Network), introduced by Noah Hollmann et al. [12], is a transformer-based network that performs supervised classification on small tabular datasets in under one second without the need for hyperparameter tuning. By training on mix proportion parameters and experimental strength data, these models can effectively capture complex relationships between components, thereby enabling high-precision predictions of concrete compressive strength.

Previous studies have generally lacked comprehensive datasets and have been limited in both scope and quality [13]. Many of these datasets are relatively small, which limits the reliability and precision of predictive models. In this study, the effects of multiple factors on concrete compressive strength were comprehensively investigated. These factors include raw material composition, physical and chemical properties, curing conditions, and other relevant parameters. To analyze these influences, a variety of modeling techniques were applied, including classical linear regression, polynomial regression, and tree-based machine learning algorithms such as Decision Tree, Random Forest, ExtraTrees, AdaBoost, CatBoost, and XGBoost, as well as the recently developed TabPFN model. In addition to the methods applied in this study, other modern machine learning techniques, such as deep learning, graph neural networks, and hybrid models, have shown promise in materials science for capturing complex, high-dimensional relationships. The selection of tree-based models and TabPFN in this work was motivated by their proven performance on tabular datasets, particularly their interpretability and computational efficiency, which make them well-suited for multicomponent concrete prediction tasks. Compared to state-of-the-art approaches, these models provide a balance between predictive accuracy and practical applicability. The primary aim of this research is to establish a robust and interpretable framework for predicting the compressive strength of multicomponent concrete, thereby addressing the limitations of conventional mix design approaches. The contribution of this work lies in advancing the application of data-driven methods in Civil Engineering, providing practical tools for optimizing mixture design and enhancing material performance in sustainable infrastructure development.

Model interpretation techniques, such as Shapley Additive exPlanations (SHAP), were employed to analyze the influence of key features on concrete compressive strength. These methods facilitate a detailed assessment of both individual and combined effects of critical variables [14]. Specifically, the interdependencies between compositional factors and material characteristics were examined to elucidate their joint impact on concrete strength. This SHAP-based analysis provides new insights into the complex relationships among features and offers valuable guidance for the design and optimization of concrete mixtures in practical engineering applications.

## 2. Materials and Methods

### 2.1. Data Pretreatment

#### 2.1.1. Test Protocol and Standard

All experimental data were obtained from complete laboratory records of concrete testing conducted across multiple regions in China. Specifically, the data were collected from the following sources: (1) Huqiang Ready-Mixed Concrete Co., Ltd. in Anyang, Henan Province (February to November 2023); (2) Nanle Concrete Laboratory in Puyang, Henan Province (June to December 2022 and March to June 2023); (3) Yonggu Concrete Laboratory in Liaocheng, Shandong Province (June to December 2022 and February to June 2023); and (4) Yanggu Concrete Laboratory in Liaocheng, Shandong Province (March to June 2023). The mean annual temperature, precipitation, and relative humidity in the sampled regions range from −14 °C to 40.8 °C, 600 to 1005 mm, and 58% to 70%, respectively. Since the data were collected exclusively in China, certain limitations may exist. Nevertheless, all sample preparation, curing, and testing in this study strictly adhered to the widely adopted method of compressive strength testing of standard-cured concrete cubes. The governing standard is Test Methods for Physical and Mechanical Properties of Concrete (GB/T 50081-2019) [15], which specifies detailed procedures covering specimen preparation, curing temperature and humidity conditions, and compressive strength testing. Standard specimens are 150 mm × 150 mm × 150 mm cubes, cured under standard conditions (Clauses 5.2.2 and 5.2.3), i.e., at 20 °C ± 2 °C and relative humidity of not less than 95%. The corresponding strength acceptance and evaluation follow the Standard for Inspection and Evaluation of Concrete Strength (GB/T 50107-2010) [16].

#### 2.1.2. Data Description

The dataset was first stripped of missing values, erroneous characters, sample identifiers and geographic variables, leaving 9998 valid records. Outlier removal (robust z-score = 3.5 on log-transformed features) was performed within each CV training fold, eliminating on average 4.2% of the samples; no points were discarded before data splitting, ensuring the test set remained unseen during cleaning.

**Figure 1:**
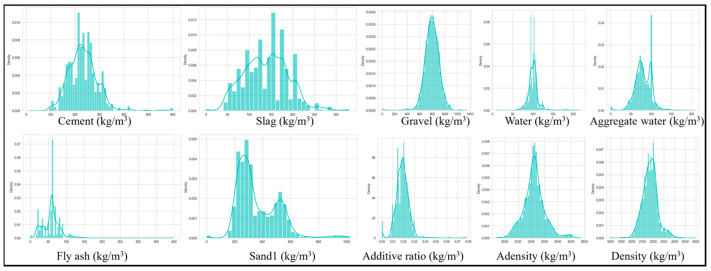
The detailed distributions of key variables.

**Figure A1:**
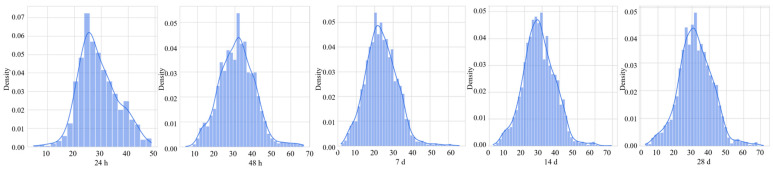
The detailed distribution of the target variable (compressive strength in different age).

**Figure A2:**
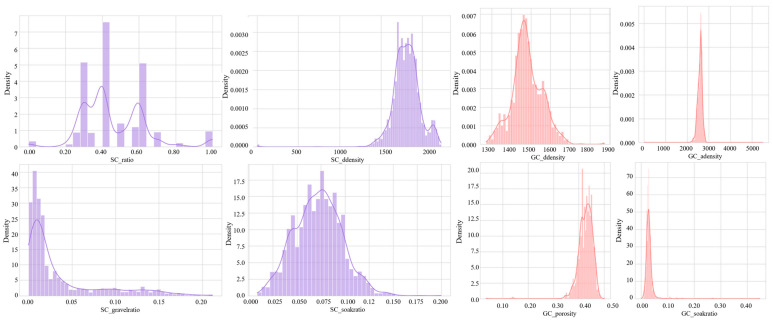
The detailed distributions of other variables.

Relevant features and target variables were identified and compiled through a manual extraction process to ensure accuracy and relevance. The final dataset comprises 22 features. Among them, acceleration curing serves as a manually assigned label, and strength is defined as the target variable. The remaining variables are treated as input features and are structured in a standardized format for analysis. The abbreviations, units, value ranges, and standard deviations of all variables are summarized in Table 1. Additionally, the 25th percentile (Q1), median (Q2), and 75th percentile (Q3) of each variable are reported. Figure 1 illustrates the detailed distributions of key variables, including cement content, slag content, fly ash content, coarse sand content, fine sand content, and stone aggregate content. The distributions of all parameters are presented in the Appendix B (Figure A1 and Figure A2).

Notably, label variables were added to distinguish whether the samples were subjected to accelerated or standard conditioning. Typically, samples under accelerated conditioning achieve high strength within a shorter time frame, while those under standard conditioning take longer to reach comparable strength levels. This approach improves generalization by preventing misclassification and overfitting.

In machine learning, using overly small test sets can lead to overestimated performance metrics that do not generalize well, whereas excessively large test sets reduce the data available for training and may negatively affect model accuracy. To balance reliability and predictive performance, a total of 9998 data points were randomly split into training and testing subsets, with the split performed prior to any preprocessing to prevent data leakage. This partitioning strategy aligns with established practices to ensure robust model validation. To investigate the impact of data preprocessing on machine learning, the original dataset (denoted X0) was standardized and normalized, producing two additional datasets labeled X1 and X2. These datasets were subsequently used for further analysis.

#### 2.1.3. Correlation Analysis

**Figure 2:**
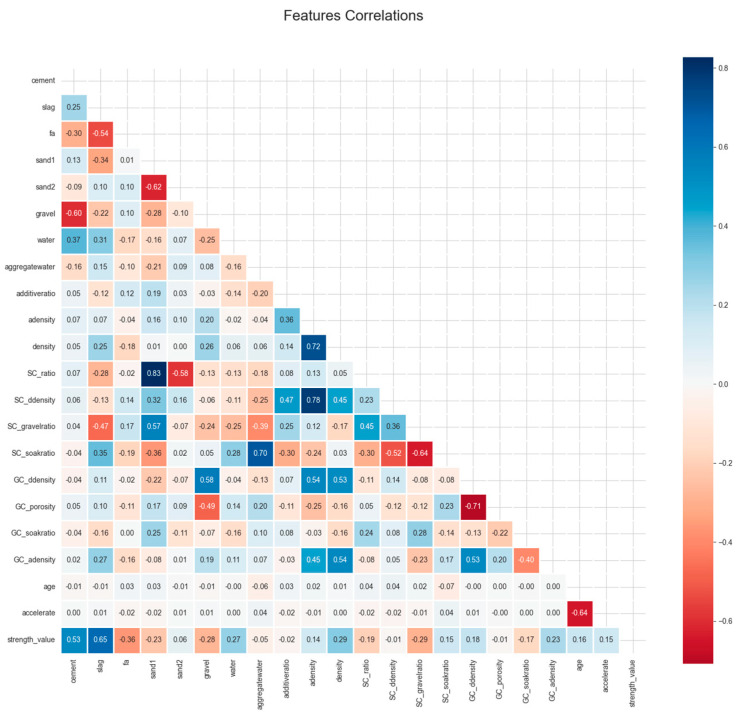
Correlation coefficient graph of the multicomponent concrete dataset.

**Figure A3:**
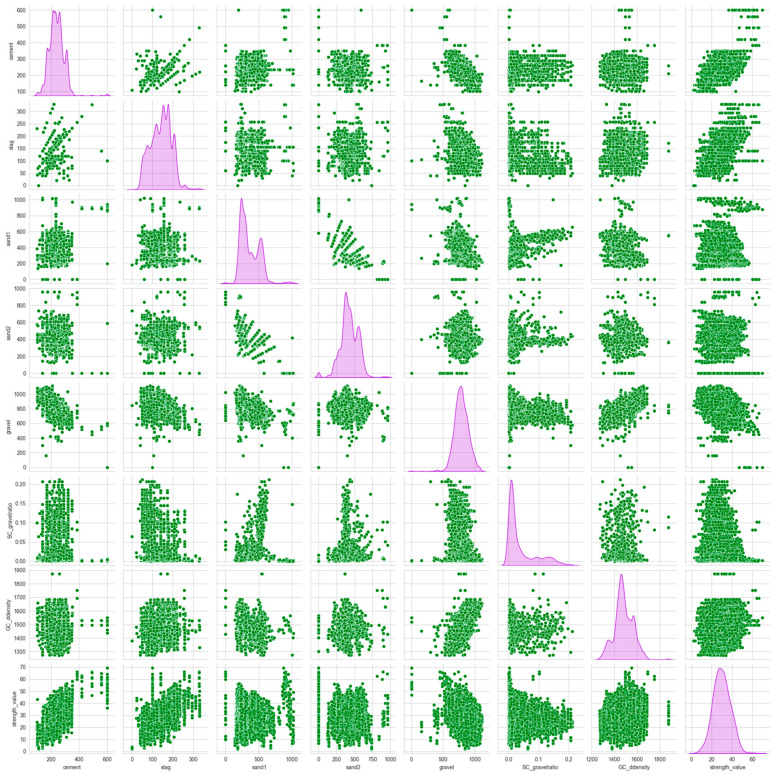
Major correlations.

As shown in Figure 2, the Pearson correlation coefficient was used to assess the relationships among the input features and between each feature and the target variable in the concrete dataset. The resulting heatmap uses a color gradient to indicate that the absolute correlation coefficient approaches one, reflecting increasingly strong positive or negative correlations between variables. Through correlation analysis, eight features were identified with absolute Pearson correlation coefficients ranging from 0.53 to 0.65, indicating moderate-to-strong correlations with the target variable. The distributions of these strongly correlated features are presented in the Appendix B (Figure A3, major correlations). Specifically, the correlation coefficient between the cement content and strength was 0.5325, and that between the slag content and strength was 0.6460, both of which suggest a significant positive correlation with the concrete strength. Additionally, a strong negative correlation was observed between GC_ddensity and GC_porosity, with a coefficient of −0.7076, indicating that aggregate bulk density and porosity are inversely related. The coefficient of 0.5272 between GC_ddensity and GC_adensity further implies a significant positive relationship between aggregate bulk density and apparent density.

Strong linear correlations among variables can undermine the stability of regression models by inflating the variance of coefficient estimates and hindering meaningful interpretation. This issue, known as multicollinearity, poses a significant challenge in predictive modeling. A more detailed discussion of this phenomenon follows in the subsequent sections.

### 2.2. Modeling

#### 2.2.1. Multicollinearity

The variance inflation factor (VIF) is a widely used metric for detecting multicollinearity, a condition in which predictor variables in a regression model are highly correlated with each other. When multicollinearity is present, it can result in unstable regression coefficients, hinder the interpretability of the model, and reduce its generalizability [17]. Therefore, the VIF is especially important when interpretable models, including linear regression, logistic regression, and generalized linear models, are constructed.

The VIF quantifies the extent to which the variance of a regression coefficient is inflated due to multicollinearity. For a given variable Xi, the VIF is calculated by regressing Xi against all other predictors and computing the coefficient of determination $R_{i}^{2}$. As shown in Equation (1):

(1) $$VIF_{i}\;=\frac{1}{1-R_{i}^{2}}$$

A higher $R_{i}^{2}$ value indicates a stronger linear relationship between a given variable and the other predictors, resulting in a higher VIF. Specifically, a VIF of 1 indicates no multicollinearity.

For example, the curing ages expressed in days (e.g., 7, 14, and 28-day) and hours (e.g., 168, 336, and 672 h) represent the same underlying variable and are highly correlated. Including both in the model adds no informational value and introduces multicollinearity. To avoid redundancy and enhance interpretability, such variables were excluded prior to model training. By removing features with excessively high VIF values, the model becomes more stable, interpretable, and better suited for generalization.

As shown in Table A1, a VIF analysis was conducted to ensure model robustness and reduce feature redundancy. The results indicate that the correlations among standardized variables fall within acceptable levels for machine learning applications. Notably, the additive ratio had the lowest VIF value of 1.38, suggesting that it is minimally influenced by other variables. In contrast, GC_density presented the highest VIF value of 35.73, indicating a high degree of multicollinearity and sensitivity to other predictors. In the subsequent analysis, the original, standardized, and normalized datasets were provided to the machine learning models to observe and compare their performance.

#### 2.2.2. Linear and Polynomial Regression

To investigate the influence of input variables on the target variable and establish a robust predictive model, this study first employs two fundamental regression methods: multiple linear regression and multiple polynomial regression [18].

In multiple linear regression, the prediction function takes the following Equation (2):

(2) $$\bar{Y}=\beta_{0}\;+\beta_{1}\;x_{1}\;+\beta_{2}\;x_{2}\;+\cdots +\beta_{p}\;x_{p}\;$$

where $\bar{Y}$ is the predicted value; $x_{1}$, $x_{2}$, …, $x_{p}$ are the independent variables; $\beta_{p}$ is the vector of regression coefficients; and $\beta_{0}$ is the intercept term (which can be considered a constant).

In multiple polynomial regression, the prediction function takes the following Equation (3):

(3) $$\bar{Y}=\beta_{0}\;+\beta_{1}x_{1}+\beta_{2}\;x_{2}\;+\ldots +\beta_{n}\;x_{n}\;+\beta_{n}\;x_{n}\;+\beta_{11\;}x_{1}^{2}\;+\beta_{12}\;x_{1}\;x_{2}\;+\ldots +\beta_{mn}\;x_{m}^{p}\;x_{n}^{q}\;+\epsilon$$

where $\bar{Y}$ is the predicted value; $x_{1}$, $x_{2}$, …, $x_{n}$ are the independent variables; $\beta_{0}\;$, $\beta_{1}$, …, $\beta_{n}\;$ are the model coefficients, including those for interaction terms and higher-order terms; p and q denote the degree of the polynomial; and ϵ is the error term vector, which represents the variation not explained by the model.

These models serve as foundational structures and can be combined with optimization strategies such as regularization. To improve generalizability and stability, four commonly used regression methods have been adopted [19]: ordinary least squares (OLS), ridge regression, least absolute shrinkage and selection operator (Lasso), and elastic net regression. OLS minimizes the sum of squared residuals and is suitable when predictors are not strongly correlated. Its objective function is Equation (4). To mitigate multicollinearity, Ridge introduces L2 regularization, and its objective function becomes Equation (5). Lasso applies L1 regularization to perform feature selection, and its objective function is given by Equation (6). Elastic Net combines both L1 and L2 penalties, providing enhanced stability and variable selection, which is particularly effective in high-dimensional settings with correlated features. The objective function for the elastic net is given by Equation (7).

(4) $$min_{\beta }\;\sum_{i=1}^{n}\;{\left(y_{i}\;-x_{i}^{⊤}\;\beta \right)}^{2}$$

(5) $$min_{\beta }\sum_{i=1}^{n}\;{\left(y_{i}\;-x_{i}^{⊤}\;\beta \right)}^{2}+\lambda \sum_{j=1}^{p}\;\beta_{j}^{2}\;$$

(6) $$min_{\beta }\;\sum_{i=1}^{n}\;{\left(y_{i}\;-x_{i}^{⊤}\;\beta \right)}^{2}+\lambda \sum_{j=1}^{p}\;|\beta_{j}\;|$$

(7) $$min_{\beta }\;\sum_{i=1}^{n}\;{\left(y_{i}\;-x_{i}^{⊤}\;\beta \right)}^{2}+\lambda (\alpha \sum_{j=1}^{p}\;|\beta_{j}\;|+(1-\alpha )\sum_{j=1}^{p}\;\beta_{j}^{2}\;)\in$$

Here, $x_{i}$ is the feature vector of the *i*-th sample, β is the vector of regression coefficients, and $y_{i}$ is the response variable in Equation (4). Here, λ ≥ 0 is the L2 regularization parameter, which controls the degree of penalty applied to the coefficients n in Equation (5). Here, λ is the L1 regularization parameter in Equation (6). Here, α is the L1 regularization parameter in Equation (6). Here, α ∈ [0, 1] controls the ratio between the L1 and L2 penalties, with α = 1 reducing to Lasso and α = 0 to Ridge in Equation (7). The L2 penalty reduces the coefficient magnitude to address multicollinearity but lacks feature selection, whereas Lasso enables variable selection by shrinking some coefficients to zero.

These regression models help compare regularization strategies and assess their suitability for predicting multicomponent concrete properties. Linear models suit small datasets with few features, whereas tree-based models work better for large, high-dimensional data.

#### 2.2.3. Tree-Based Modeling

Tree-based methods have strong nonlinear fitting capabilities, flexible structures, and good generalization performance, making them important modeling tools for predicting the performance of complex materials such as multicomponent concrete [20]. To further enhance the nonlinear modeling ability and generalization performance of regression models, this work introduces tree-based regression methods (Decision Tree, Random Forest, ExtraTrees, AdaBoost, CatBoost, and XGBoost). These methods can capture complex interactions between features, offering strong interpretability and robustness, especially in scenarios where nonlinear relationships or higher-order interactions exist between variables. They are widely used in engineering modeling and complex system prediction tasks.

(1) Decision tree regressor

Decision tree regression is a nonparametric model that partitions the feature space into multiple subregions via a series of “if-then” rules and outputs a constant value within each subregion. The splitting process is based on the feature values of the samples, and the optimal partition is determined by minimizing the mean squared error (MSE) at the leaf nodes. A single decision tree suffers from high variance and overfitting, despite its interpretability.

(2) Random forest regressor

To address the high variance of a single tree, random forests consist of multiple independently trained decision trees. The model improves generalizability by incorporating feature subset selection (feature bagging) and sample resampling (bootstrap sampling). The final prediction is the average output of all the trees, which effectively reduces overfitting and significantly enhances both stability and accuracy.

(3) Extra trees regressor

The extra trees regressor is an ensemble learning method based on decision trees. Unlike traditional decision trees, it introduces more randomness by using the original training set without bootstrap sampling. For feature selection, it randomly selects a feature value rather than using information gain or the Gini index, which helps reduce variance but may increase bias.

Compared with the random forest, the ExtraTrees regressor may offer better generalization in some cases, as its trees tend to be larger, thus reducing variance. Additionally, its purely random partitioning often results in faster training speeds. While it is not as commonly used as decision trees or random forests are, it remains a valuable tool, especially in scenarios requiring greater randomness and faster training.

(4) Adaptive boosting regressor (AdaBoost)

AdaBoost is a stepwise weighted ensemble learning method. Its core idea is to train a new weak learner in each iteration and adjust the sample weights based on the prediction errors of the previous model, allowing the model to focus more on the previously misclassified samples. The final model output is the weighted sum of multiple weak learners, which enhances its adaptive modeling ability.

(5) CatBoost regressor

CatBoost, developed by Yandex [21], is a gradient boosting framework optimized for handling categorical features. Its regression model, the CatBoost regressor, builds and combines a series of weak learners (usually decision trees) to improve prediction accuracy and robustness. CatBoost employs target encoding and ordering enhancement mechanisms that prevent information leakage, effectively enhancing the model’s ability to handle categorical features. It is less sensitive to parameter changes, offers fast training speeds, and delivers excellent accuracy.

(6) XGBoost regressor

XGBoost is an efficient gradient boosting framework that is particularly well suited for handling large-scale data and sparse features. It enhances traditional gradient boosting methods by incorporating second-order gradient optimization, custom regularization terms, parallel training, and automatic handling of missing values. These mechanisms not only improve training efficiency but also effectively control model complexity, enhancing both generalization ability and stability.

These models, ranging from single trees to ensemble methods, progressively enhance model capability. They offer powerful nonlinear modeling abilities while also addressing feature selection and error control, providing a rich set of options and references for subsequent modeling and comparison.

#### 2.2.4. TabPFN

In January 2025, Noah et al. published a study in Nature on accurate predictions of small data with a tabular foundation model, introducing TabPFN (Tabular Prior-data Fitted Network), a transformer-based model for tabular data. TabPFN uses an end-to-end framework with a large transformer encoder pretrained through meta-learning on synthetic tasks [12]. This allows the model to effectively predict target outcomes from limited training data.

The TabPFN is suitable for small-sample, low-dimensional tasks and requires little to no hyperparameter tuning, although it is sensitive to the data distribution. By elevating regression modeling to the level of distributional prediction, TabPFN exhibits strong generalizability and robustness, showing great potential for applications in engineering domains.

Given the challenges posed by small datasets, complex nonlinear relationships, and high noise levels in concrete property prediction, TabPFN offers a probabilistic modeling approach that shifts from point prediction to distribution prediction. This method not only improves robustness in uncertainty-sensitive scenarios but also allows for a more nuanced understanding of the predictions. In this study, the TabPFN is applied to concrete composition and property prediction, and it is compared with traditional deterministic models.

### 2.3. Interpretation

The SHapley additive exPlanations (SHAP) algorithm is a widely used post hoc interpretation tool that applies to machine learning models and is notably efficient for tree-based algorithms [22]. It is employed after training and produces contribution values so that each prediction can be expressed as a fixed base value (equivalent to the mean target value in regression) plus the aggregated contributions of individual features for model prediction Equation (8):

(8) $$f(x)=f_{\mathrm{base}}+\sum_{i=1}^{n}\phi_{i}(x)$$

where $f_{\mathrm{base}}$ is the base value (the mean target value for regression models) and where $\sum_{i=1}^{n}\phi_{i}(x)$ is the SHAP value for feature *i*, indicating its marginal contribution. This formulation, rooted in cooperative game theory, ensures that each feature’s influence is precisely quantified.

### 2.4. Evaluation Indicators

To evaluate model performance, four evaluation metrics were employed [23]. Equation (9) defines the mean absolute error (MAE), which averages the absolute differences between the predicted and actual values. Equation (10) describes the mean squared error (MSE), emphasizing larger discrepancies by squaring the errors. Equation (11) provides the root mean squared error (RMSE), obtained by taking the square root of MSE so the unit matches the observed values. Finally, Equation (12) introduces the coefficient of determination (R^2^), which is calculated as 1 minus the ratio of residual to total variance, where values closer to 1 indicate a better fit.

(9) $$\mathrm{MAE}={\frac{1}{n}\sum }_{i=1}^{n}\left|y_{i}-{\overset{`}{y}}_{i}\right|$$

(10) $$\mathrm{MSE}={\frac{1}{n}\sum_{i=1}^{n}\left(y_{i}-{\overset{`}{y}}_{i}\right)}^{2}$$

(11) $$\mathrm{RMSE}=\sqrt{\frac{\sum_{i=1}^{n}{\left(y_{i}-{\overset{`}{y}}_{i}\right)}^{2}}{n}}$$

(12) $$R^{2}=1-\frac{\sum_{i=1}^{n}{\left(y_{i}-{\overset{`}{y}}_{i}\right)}^{2}}{\sum_{i=1}^{n}{\left(y_{i}-\overset{⃐}{y}\right)}^{2}}$$

## 3. Results and Discussion

### 3.1. Prediction of the ML Models

In the context of concrete performance prediction and functional optimization, there is no universally optimal algorithm. Selecting the most appropriate model typically requires iterative experimentation and comparative evaluation. In this study, eight supervised learning algorithms were assessed, and their predictive performance was significantly enhanced through systematic hyperparameter tuning. The optimal hyperparameters were identified via GridSearchCV from the scikit-learn library, which efficiently automates the search process and reduces the computational burden. Each model was trained on well-prepared datasets, with hyperparameters optimized via 8-fold cross-validation to ensure robust and reliable performance estimates. The final configurations, selected based on average validation metrics, led to improved accuracy and generalizability across models.

#### 3.1.1. Linear and Polynomial Prediction

**Figure 3:**
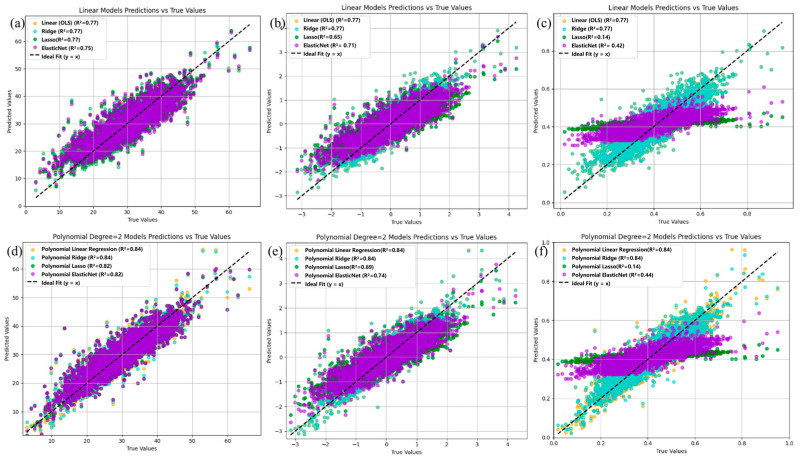
The prediction results of linear regression on (**a**) X0, (**b**) X1, and (**c**) X2 and polynomial regression on (**d**) X0, (**e**) X1, and (**f**) X2.

Classical multiple linear regression and multiple polynomial regression models are employed to predict the potential relationships between the concrete strength and 22 features. Figure 3a,d displays the prediction results of OLS regression and polynomial regression, as well as their fitted results via ridge regression, Lasso regression, and elastic net regression, which are applied to the concrete characteristic dataset X0.

Figure 3a shows that the R^2^ results of the test set (0.75–0.77) indicate that OLS effectively identifies the relationship between the features and concrete strength. Moreover, applying ridge, Lasso, and elastic net regression methods to the linear model on dataset X0 did not result in significant differences in the fitting results. The corresponding polynomial fitting predictions are shown in Figure 3d, where R^2^ ranges from 0.82 to 0.84, suggesting that polynomial fitting outperforms linear fitting. Although the three regularization methods yielded similar results, they may lead to better generalization, potentially offering improved performance on more complex datasets.

Figure 3b,e shows the prediction results of linear regression and polynomial regression, along with the regularization methods, on the standardized dataset X1. The predictive performance of the base models remains largely unchanged. However, the performance of the regularized models slightly decreases, particularly in the case of Lasso regression, where the R^2^ values decrease to 0.65 and 0.71, respectively. This effect becomes more pronounced on the normalized dataset X2, as illustrated in Figure 3c,f. This may be attributed to the fact that linear and polynomial regression are generally insensitive to feature scaling [24], as their objective is solely to minimize the sum of squared errors without any regularization term. Consequently, whether features are standardized or normalized does not affect the relative proportions of the estimated parameters. In contrast, Lasso and Elastic Net are sensitive to feature scaling, and their performance depends more heavily on careful tuning of the regularization parameter to achieve optimal results.

#### 3.1.2. Tree-Based Model Prediction

**Figure 4:**
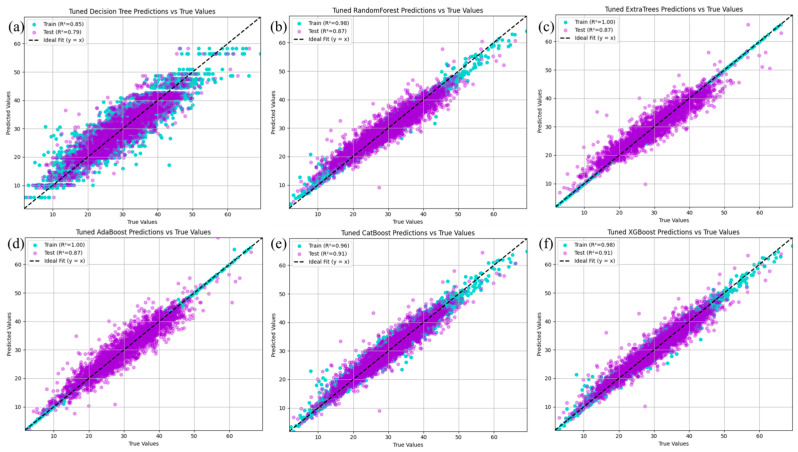
The prediction results of (**a**) decision trees, (**b**) random forests, (**c**) extratree regressors, (**d**) AdaBoostRegressor, (**e**) CatBoostRegressor, and (**f**) XGBRegressor.

Tree-based models offer strong nonlinear fitting capabilities, structural flexibility, and good generalization performance, making them effective tools for predicting the properties of complex materials such as multicomponent concrete [25]. To further enhance the performance of machine learning models, this study introduces six representative tree-based regression methods. Compared with OLS and polynomial regression, tree-based models are less sensitive to data preprocessing [26]. Accordingly, the three datasets X0, X1, and X2 were all used for model fitting, and the best-performing results are presented in Figure 4.

The results from the decision tree model show that it captured the relationship between features and concrete strength in the training set, with an R^2^ of 0.87. However, its generalizability was limited, as the R^2^ on the test set decreased to 0.79. To improve the predictive performance, a random forest was applied, resulting in a significant increase in R^2^ to 0.98 on the training set and 0.87 on the test set.

Notably, models such as the ExtraTrees regressor and AdaBoost regressor exhibited overfitting. Although their R^2^ values on the test set reached 0.87, both achieved a perfect fit (R^2^ = 1) on the training set, suggesting potential overfitting.

Subsequently, the CatBoost Regressor and XGB Regressor were used to learn the relationships between features and concrete strength. Both models showed strong performance, with R^2^ values of 0.96 and 0.98 on the training set, and 0.91 on the test set, respectively, demonstrating both high predictive accuracy and good generalization ability.

#### 3.1.3. TabPFN Prediction

This study further applied a recently reported ML algorithm, TabPFN, which has strong generalizability and robustness, particularly on small-sample datasets [12], and shows promise in engineering applications [27]. However, the concrete dataset used in this study contains 9998 samples and 22 features, making it relatively large dataset. To better align with the strengths of TabPFN, the dataset was partitioned into five smaller subsets based on curing age.

**Figure 5:**
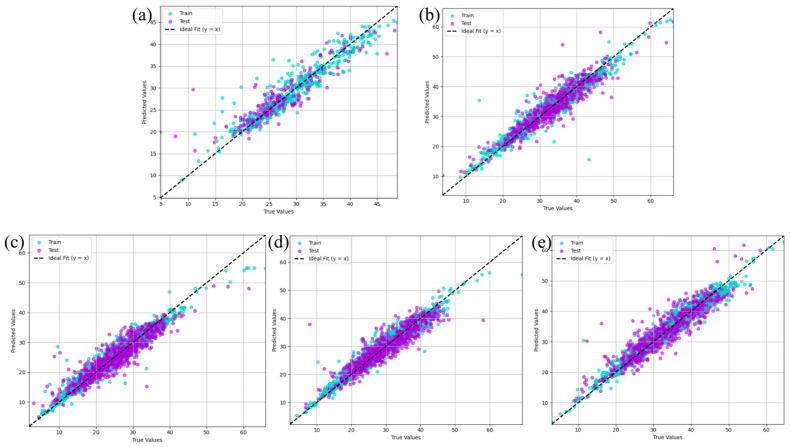
The prediction results of TabPFN on five subsets. (**a**) 24 h steam curing, (**b**) 48 h steam curing, (**c**) 7 d standard curing, (**d**) 14 d standard curing, (**e**) 28-day standard curing.

Five subsets were created according to the curing time, with average strengths of 28.69 MPa (24 h steam curing), 32.90 MPa (48 h steam curing), 24.83 MPa (7 d standard curing), 29.49 MPa (14 d standard curing), and 33.30 MPa (28-day standard curing). TabPFN was then used for fitting and prediction on each subset. Each subset was also standardized and normalized, and model fitting was performed on both versions. The best results are presented in Figure 5.

Figure 5a shows that the compressive strength of 24 h steam-cured concrete ranges from 5 to 50 MPa, with most values concentrated between 15 and 45 MPa. Despite having only 687 samples, the model effectively captured the relationship between material features and strength, achieving an R^2^ of 0.89. Figure 5b presents the results for the 48 h curing dataset, where the model also successfully identified the underlying patterns. Although a few outliers are present, the overall prediction reached an R^2^ of 0.91.

Figure 5c–e displays the predictions for 7-day, 14-day, and 28-day standard cured concrete. The model achieved R^2^ values of 0.91, 0.91, and 0.92, respectively. These results indicate that TabPFN effectively captures the relationships between features and strength, delivering reliable predictions even on smaller datasets without signs of overfitting or underfitting.

### 3.2. Performance of the ML Models

#### 3.2.1. Linear and Polynomial Fit

**Figure 6:**
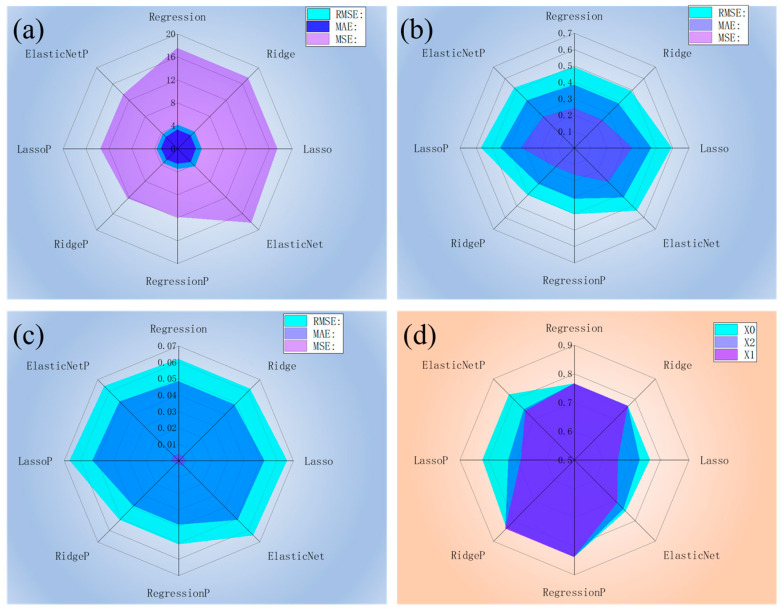
Model evaluation metrics, including the MSE, RMSE, and MAE, on (**a**) the original dataset X0, (**b**) the standardized dataset X1, and (**c**) the normalized dataset X2. (**d**) R^2^ of each dataset.

To evaluate model performance comprehensively, four metrics are used: R^2^, MSE, RMSE, and MAE. R^2^ measures the explained variance, MSE and RMSE assess the overall prediction error, and MAE reflects the average prediction bias [28]. For both the linear and polynomial regression models, the performance difference between the training and test sets is minimal. Therefore, only test set results are presented to better reflect real performance. Figure 6a–c illustrates the model evaluation metrics, including the MSE, RMSE, and MAE, on the original dataset X0, the standardized dataset X1, and the normalized dataset X2, respectively.

As shown in Figure 6a, all the linear and polynomial models performed poorly on the original dataset X0. Polynomial regression generally outperforms linear models. Notably, the linear model processed with elastic net regression produced the poorest MSE, reaching 18.34.

As shown in Figure 6b, all the linear and polynomial models on the standardized dataset yielded R^2^ values less than 0.6. Among them, ridge regression and standard polynomial regression performed best, with nearly identical MSE, RMSE, and MAE values of 0.16, 0.41, and 0.40, respectively. In Figure 6c, all the linear and polynomial models on the normalized dataset achieved significantly lower errors, with RMSE and MAE values below 0.07 and even smaller MSE values.

However, these lower error metrics do not necessarily indicate better model performance. The observed reduction is due primarily to normalization scaling the target variable (concrete strength) from 0 to 60 MPa to 0–1. As a result, the prediction errors shrink proportionally, even if the model quality remains unchanged. Therefore, when comparing model performance across datasets, R^2^ is a more reliable metric since it is scale independent. From this perspective, dataset X0 already provides sufficient accuracy and good generalization ability, and further improvements in accuracy can be achieved by using dataset X2.

As shown in Figure 6d, the performance of the linear and polynomial models across the three datasets is relatively consistent. The fitting results are symmetric along the diagonal, with polynomial regression outperforming linear regression under the same conditions. For example, on the standardized dataset, both the Lasso-regularized linear and polynomial models produce lower R^2^ values, with the polynomial model achieving slightly higher predictive accuracy. These findings suggest that, for the present dataset, polynomial regression on the original dataset provides more accurate fitting and prediction.

#### 3.2.2. Tree-Based Model Fit

**Figure 7:**
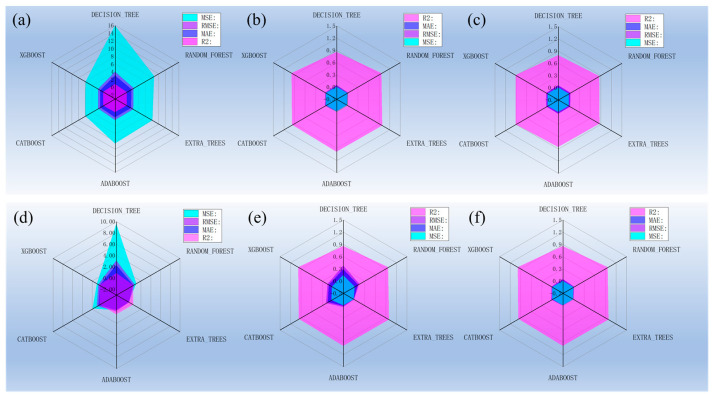
Fitting results of the six tree models on the three datasets for both test sets (**a**) original dataset X0, (**b**) standardized dataset X1, and (**c**) normalized dataset X2 and training sets (**d**) original dataset X0, (**e**) standardized dataset X1, and (**f**) normalized dataset X2.

Differences were observed between the training and test set results for the models. Figure 7 shows the fitting results of the six tree models on the three datasets for both the training and test sets. As shown in Figure 7a,d, the fitting results for all six models on the original dataset are relatively poor, with the decision tree model exhibiting MSE values of 10.00 and 16.00 on the training and test sets, respectively. This issue is resolved after standardization. Figure 7b,e shows improved fitting results on the standardized dataset, with all the models achieving optimal performance on both the training and test sets. Notably, after standardization, the models yield better results on the test set than on the training set, indicating strong generalizability. Figure 7c,f presents the fitting results on the normalized dataset, where the models also perform well on both sets. Compared with linear and polynomial regression models, tree models, especially the random forest, ExtraTrees regressor, AdaBoost regressor, CatBoost regressor, and XGB regressor, yield significant improvements, with R^2^ values exceeding 0.9 and low values for MSE, RMSE, and MAE. Among them, XGBoost achieves the best overall performance across all datasets. Therefore, when using tree-based models, dataset X2 outperforms X1, which in turn outperforms X0.

#### 3.2.3. TabPFN Model Fit

**Figure 8:**
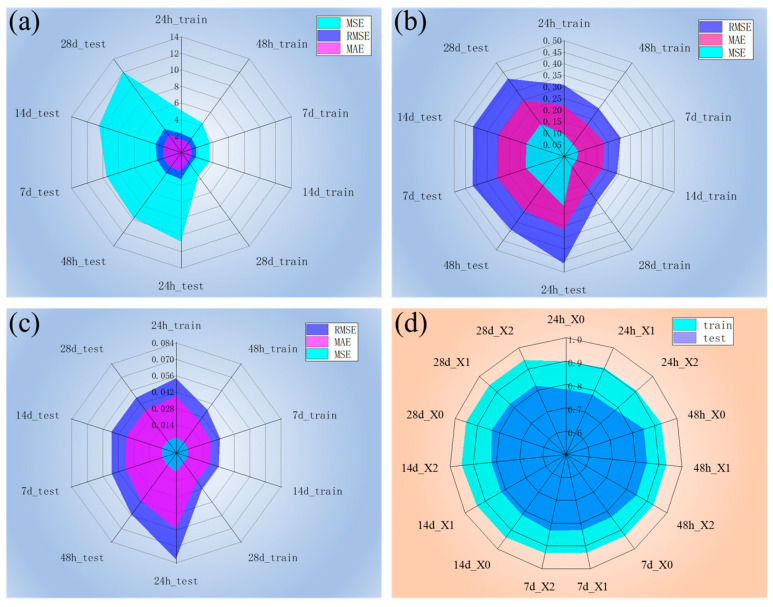
TabPFN model evaluation metrics, including the MSE, RMSE, and MAE, on (**a**) the original dataset X0, (**b**) the standardized dataset X1, and (**c**) the normalized dataset X2. (**d**) R^2^ of each dataset.

Figure 8 presents the TabPFN model performance across all datasets. The model performance of the TabPFN improves significantly after data standardization. As shown in Figure 8b, the 28-day test set achieves MSE, RMSE, and MAE values of 0.17, 0.41, and 0.30, respectively, which are substantial improvements over the original dataset results (MSE = 11.98, RMSE = 3.46, MAE = 2.57 in Figure 5a). A similar trend is observed for the 24 h dataset, where the metrics decrease from 10.84, 3.29, and 2.25 to 0.22, 0.47, and 0.32, respectively, after standardization.

The model performance further improves on the normalized datasets. As shown in Figure 8c, the 28-day and 24 h test sets yield MSE, RMSE, and MAE values of 0.002, 0.05, and 0.035, and 0.007, 0.08, and 0.056, respectively. Figure 8d shows the R^2^ values across all datasets. Except for the 24 h accelerated curing subset, which shows slightly lower performance (approximately 0.90 on the training set and 0.78 on the test set), all other subsets maintain high R^2^ values (above 0.93 for training and 0.82 for testing). Compared with tree-based models, the TabPFN achieves comparable predictive accuracy on significantly smaller datasets, demonstrating strong efficiency and generalizability. Therefore, for the TabPFN model, based on the MSE, RMSE, and MAE values, dataset X2 outperforms X1, which in turn outperforms X0. It is worth noting that according to the R^2^ values, there is no significant difference in accuracy among all datasets. The accuracy of the prediction results for each method, including the 28-day strength validation on the original dataset, is summarized in Table A2.

### 3.3. Feature Analysis

#### 3.3.1. Feature Impact on the Concrete Strength

**Figure 9:**
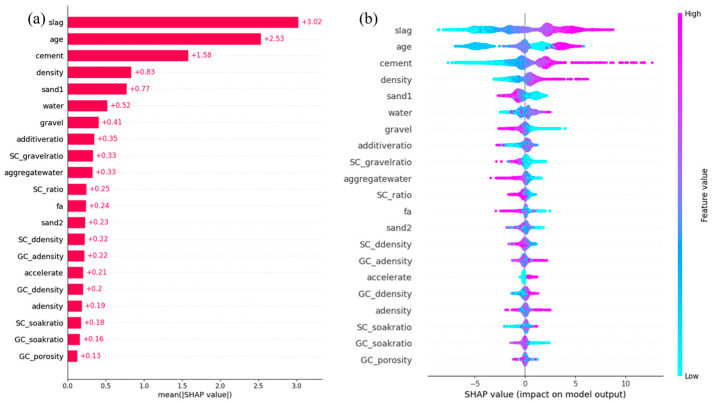
Interpretation of the optimal (XGB) model using SHAP (Shapley Additive exPlanations). (**a**) Mean absolute SHAP values, representing the global importance of each feature. (**b**) Beeswarm plot of SHAP values for each feature. The color represents the feature value (from low in blue to high in red), and the horizontal position shows the impact of that value on the model’s prediction (negative impact for SHAP values < 0, positive impact for SHAP values > 0). Features are ordered descending by their mean absolute SHAP value.

To gain deeper insight into the decision-making mechanism of the model and the contribution of each input feature, Shapley Additive exPlanations (SHAP) analysis was applied to the optimal model (XGB) trained on the original dataset. SHAP values provide local interpretability for individual predictions and quantify global feature importance by averaging absolute values across all samples, effectively mitigating the bias introduced by feature scaling [29]. As illustrated in Figure 9a, the mean absolute SHAP values reflect the overall influence of each feature on the model output.

As shown in the figure, the top three contributing features are “slag,” “age,” and “cement.” Among them, “slag” has the highest average SHAP value of 3.02, indicating that even small variations in slag content can substantially influence concrete strength. The model also assigns high importance to “age”, which refers to the curing time (24 h, 48 h, 168 h, 336 h, and 672 h) and is known to significantly affect concrete carbonation. “Cement” ranks third, suggesting that the cement content also plays a crucial role in strength development. These findings align well with the established understanding of concrete strength evolution.

Features such as “density,” “sand1,” “water,” and “gravel” have secondary influences on concrete strength. As illustrated in Figure 9b, “density” follows a similar trend to the major features: lower values (blue bars) are associated with weaker or even negative effects, whereas higher values (red bars) positively contribute to strength. In contrast, higher contents of “sand1” and “gravel” are linked to stronger negative impacts on strength. Water generally has a positive effect, although its values are concentrated within a narrow range, to maintain consistent concrete performance.

Although the values of additives are generally low, even a small amount of additive can positively impact the concrete strength, as indicated by the red region in Figure 9b. Features such as SC_gravel ratio, aggregate water, SC_ratio, fa, and sand2 exhibit notable negative effects. While sand2 shows a similar trend to that of sand1, its influence is less pronounced, offering practical insights for concrete design.

SC_ddensity and GC_adensity have comparable levels of importance but opposite effects: GC_adensity contributes positively to strength, whereas SC_ddensity has a negative impact. This contrast may be attributed to microstructural interactions at the interface between the aggregates and cementitious material, suggesting a potential direction for future research in concrete chemistry and reaction kinetics.

Interestingly, the model successfully recognized the influence of the binary label variable acceleration (1 or 0), with accelerated curing clearly leading to better strength development than nonaccelerated curing.

Moreover, compared with that of gravel, the water absorption ratio of sand (SC_soak ratio, SHAP value = 0.18) has a stronger and opposite effect (GC_soak ratio, SHAP value = 0.16). High sand absorption appears to be beneficial for strength, whereas high gravel absorption has a detrimental impact. The influence of the aggregate void ratio is minimal (SHAP value = 0.13), and the data suggest that it predominantly contributes negatively to strength.

#### 3.3.2. Dependence and Interpretation

In real-world engineering problems, influencing variables rarely act in isolation; instead, they often interact and depend on one another. For example, in predicting concrete material properties, nonlinear coupling between components may occur. These feature interactions can significantly affect both model accuracy and interpretability [30]. Identifying and modeling such dependencies not only improves predictive performance but also helps uncover underlying physical or engineering mechanisms.

**Figure 10:**
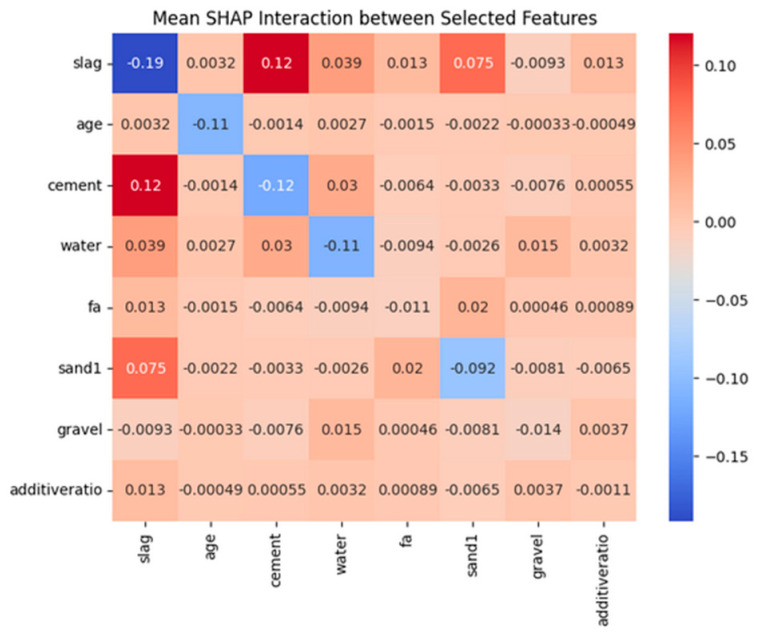
The average SHAP interaction value heatmap.

**Figure A4:**
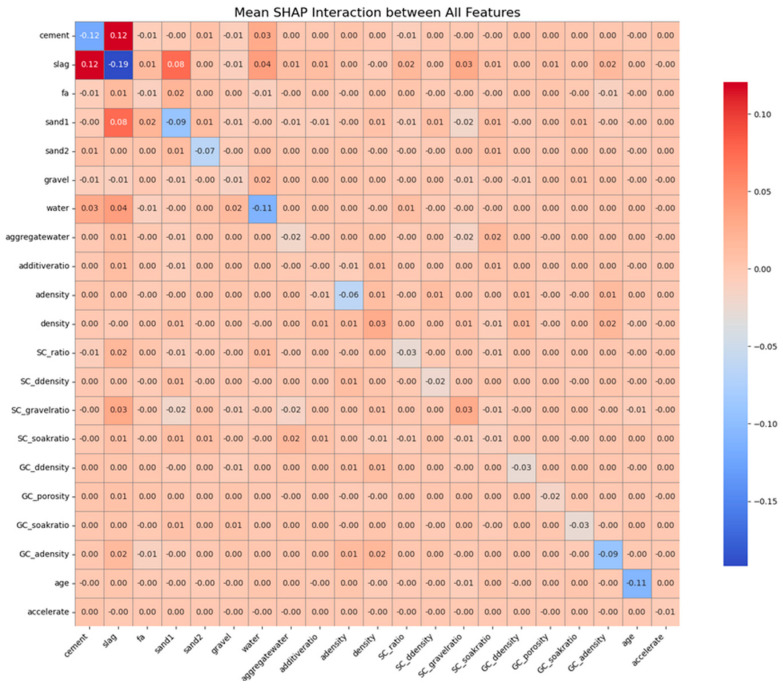
The overall feature interaction patterns.

The overall feature interaction patterns are visualized in Figure A4, revealing sparse yet meaningful feature interactions. Figure 10 highlights the interaction effects among the eight most influential features.

Figure 10 presents the average SHAP interaction value heatmap, which reveals how pairwise feature interactions contribute jointly to the model’s predictions. Each element in the matrix represents the mean SHAP interaction value between two features, with the numerical value and color indicating the strength and direction of the interaction (red indicates a positive interaction; blue indicates a negative interaction). It provides insight into how feature pairs jointly influence model output.

Notably, a strong positive interaction is observed between slag and cement (+0.12), indicating that their combined variation has a synergistic effect on concrete strength that exceeds the sum of their individual contributions. Similarly, the interaction between slag and sand1 (+0.075) suggests that the presence of fine aggregates influences the role of slag in strength development. In contrast, significant negative self-interactions are found for features such as slag (−0.19), age (−0.11), and cement (−0.12), which implies the presence of nonlinear saturation effects. Further increases in these variables may result in diminishing marginal gains in concrete strength.

Overall, this heatmap highlights the machine learning model’s ability to capture complex, nonlinear dependencies among features in a multicomponent system such as concrete. Such SHAP interaction analysis not only supports accurate prediction but also enhances interpretability, offering quantitative evidence of the underlying mechanisms governing material behavior. This contributes to advancing data-driven material design in a transparent and mechanism-informed manner.

## 4. Conclusions and Perspective

A comprehensive machine learning method has been developed based on a large concrete engineering dataset. Conclusions and perspectives are summarized below.

(1) Machine learning models significantly increase the efficiency of concrete strength prediction, reducing the experimental cost and time. Among them, tree-based models exhibit strong generalizability and accuracy, with XGBoost achieving the best overall performance across datasets (test set R^2^ = 0.91; MSE = 6.75, RMSE = 2.60, MAE = 1.91), demonstrating high robustness and practical reliability.
(2) Traditional linear and polynomial regression methods face performance limitations. Even with regularization techniques such as ridge, Lasso, and elastic net, improvements are not guaranteed. Model accuracy remains highly sensitive to regularization parameters and generally falls short of tree-based models without careful tuning.
(3) Like any empirical method, the TabPFN model also has its limitations. It performs best in small-scale, high-precision prediction tasks. When the datasets are divided by curing age and normalized, TabPFN achieves its highest accuracy on the 28-day strength test set. However, when applied to a full dataset, discrepancies between the actual and predicted results become evident.
(4) Slag, age, and cement are identified as the most influential positive features in the development of concrete strength. In contrast, higher contents of sand and aggregates have a negative impact. Notably, feature interaction analysis reveals a strong positive synergy between slag and cement (+0.12), indicating that their combined effect on strength exceeds their individual contributions.

## Figures and Tables

**Table 1:** Abbreviations, units, value ranges, and standard deviations of the dataset.

Parameter	Abbreviation	Unit	25% ^1^	50% ^1^	75% ^1^	SD ^2^
Cement content	cement	kg/m^3^	210	234	268	52.88
Slag content	slag	kg/m^3^	105	148.40	178.00	50.16
Fly ash content	fa	kg/m^3^	35.00	60.00	70.00	24.16
fine sand content	sand1	kg/m^3^	244.00	315.00	488.97	144.54
coarse sand content	sand2	kg/m^3^	358.00	420.60	521.25	122.31
Stone aggregate content	gravel	kg/m^3^	718.00	785.00	859.85	110.40
Water content	water	kg/m^3^	96.00	103.00	107.00	11.92
Aggregate water consumption	aggregatewater	kg/m^3^	65.02	74.00	86.00	18.68
Proportion of additives	additiveratio	kg/m^3^	0.01	0.01	0.02	0.01
Apparent density	adensity	kg/m^3^	2175.00	2219.00	2255.00	72.01
Actual density	density	kg/m^3^	2125.00	2170.00	2208.00	66.64
Proportion of silt	SC_ratio	-	0.30	0.40	0.60	0.15
Actual density of sand	SC_ddensity	kg/m^3^	1719.00	1816.00	1894.00	133.58
Stone content of sand	SC_gravelratio	-	0.00	0.01	0.05	0.04
Water absorption rate of sand	SC_soakratio	-	0.05	0.06	0.08	0.02
Actual density of stone	GC_ddensity	kg/m^3^	1435.00	1475.00	1538.00	80.73
Porosity of stone	GC_porosity	-	0.41	0.43	0.44	0.02
Water absorption rate of stone	GC_soakratio	-	0.02	0.02	0.03	0.01
Apparent density of stone	GC_adensity	kg/m^3^	2528.00	2603.00	2652.32	101.53
Curing age	age	h	48.00	336.00	672	231.66
Accelerating curing (label)	accelerate	-	0/1	0/1	0/1	0.44
Strength value	strength_value	MPa	23.90	29.50	35.50	8.55

^1^ Quartiles (Q1, Q2, Q3) in statistics; ^2^ Standard deviation.

## Data Availability

The original contributions presented in this study are included in the article. Further inquiries can be directed to the corresponding authors.

## Abbreviations

The following abbreviations are used in this manuscript:

TabPFN	Tabular Prior-data Fitted Network
SHAP	Shapley Additive exPlanations
SD	Standard deviation
VIF	Variance inflation factor
OLS	Ordinary least squares
MSE	Mean squared error
MAE	Mean absolute error
RMSE	Root mean squared error
R^2^	Coefficient of determination

## Appendix A

### Appendix A.1

**Table A1:** VIF analysis of variables.

Variable	VIF Value
cement	3.33
slag	3.53
fa	1.99
sand1	10.25
sand2	4.52
gravel	7.36
water	2.01
aggregatewater	3.82
additiveratio	1.38
adensity	11.01
density	2.84
SC_ratio	3.46
SC_density	7.88
SC_gravelratio	3.55
SC_soakratio	4.82
GC_density	35.73
GC_porosity	22.59
GC_soakratio	1.56
GC_adesnity	15.64
age	1.72
accelerate	1.71

### Appendix A.2

**Table A2:** Performance comparison of ML models over the mean absolute error (MAE), mean squared error (MSE), root mean squared error (RMSE), and coefficient of determination (R^2^).

Algorithm	In Training Dataset				In Testing Dataset			
	MSE	RMSE	MAE	R^2^	MSE	RMSE	MAE	R^2^
Decision trees	9.5776	3.0948	2.2985	0.8679	15.9180	3.9897	3.0279	0.7871
Random forests	1.7193	1.3112	0.9927	0.9763	9.9388	3.1526	2.4360	0.8671
Extratree regressors	0.0049	0.0699	0.0019	0.9999	9.3698	3.0610	2.3280	0.8747
AdaBoost	0.1427	0.3778	0.1141	0.9980	9.8365	3.1363	2.3697	0.8685
CatBoost	2.5916	1.6099	1.2018	0.9643	6.8385	2.6151	1.9289	0.9086
XGBoost	1.5776	1.2560	0.9289	0.9782	6.7487	2.5978	1.9131	0.9098
TabPFN	3.5752	1.8908	1.4210	0.9410	11.9858	3.4621	2.5662	0.8216

## References

[1] Li C., Chen X., Yuan C.X. (2025). Does digital government reduce carbon emissions? Empirical evidence from global sources. J. Environ. Manag..

[2] Dixit A., Du H., Dang J., Pang S.D. (2021). Quaternary blended limestone-calcined clay cement concrete incorporating fly ash. Cem. Concr. Compos..

[3] Nazeer M., Kapoor K., Singh S.P. (2023). Strength, durability and microstructural investigations on pervious concrete made with fly ash and silica fume as supplementary cementitious materials. J. Build. Eng..

[4] Wang X., Wang W., Li Y., Wang L., Duan P., Liu Y. (2025). Water absorption and desorption behavior of lightweight aggregate for internal curing in cement-based materials: A critical review. J. Build. Eng..

[5] Jiang X., Lu J.-X., Luo X., Leng Z., Poon C.S. (2025). Enhancing photocatalytic durability of high strength pervious concrete: Micro-mechanical and microscopic mechanisms. Cem. Concr. Compos..

[6] Zhang P., Cui Y., Douglas K., Song C., Russell A.R. (2025). Phase field fracture modeling of cohesive-frictional materials like concrete and rock using the scaled boundary finite element method. Comput. Geotech..

[7] Khormani M., Jaari V.R.K. (2023). Statistical analysis of the compressive strength of concrete using 2D DIP technology and Finite Element Method. Case Stud. Constr. Mater..

[8] Amrani M., El Haloui Y., Tlidi A., Barbachi M., Taha Y. (2021). A new empirical model for predicting complex modulus of asphalt concrete materials. Mater. Today Proc..

[9] Paul S.C., Panda B., Huang Y., Garg A., Peng X. (2018). An empirical model design for evaluation and estimation of carbonation depth in concrete. Measurement.

[10] Choung S., Park W., Moon J., Han J.W. (2024). Rise of machine learning potentials in heterogeneous catalysis: Developments, applications, and prospects. Chem. Eng. J..

[11] Liu X., Fan K., Huang X., Ge J., Liu Y., Kang H. (2024). Recent advances in artificial intelligence boosting materials design for electrochemical energy storage. Chem. Eng. J..

[12] Hollmann N., Müller S., Eggensperger K., Hutter F. (2025). Accurate predictions on small data with a tabular foundation model. Nature.

[13] Sarkar K., Shiuly A., Dhal K.G. (2024). Revolutionizing concrete analysis: An in-depth survey of AI-powered insights with image-centric approaches on comprehensive quality control, advanced crack detection and concrete property exploration. Constr. Build. Mater..

[14] Wang J., Zhang Z., Liu X., Shao Y., Liu X., Wang H. (2024). Prediction and interpretation of concrete corrosion induced by carbon dioxide using machine learning. Corros. Sci..

[15] (2019). Test Methods for Physical and Mechanical Properties of Concrete.

[16] (2010). Standard for Inspection and Evaluation of Concrete Strength.

[17] Wang D., Ji Y., Xu W., Lu J., Dong Q. (2025). Multi-objective optimization design of recycled concrete based on the physical characteristics of aggregate. Constr. Build. Mater..

[18] Kumari P., Paruthi S., Alyaseen A., Khan A.H., Jijja A. (2024). Predictive performance assessment of recycled coarse aggregate concrete using artificial intelligence: A review. Clean. Mater..

[19] Geng S.-Y., Luo Q.-L., Cheng B.-Y., Li L.-X., Wen D.-C., Long W.-J. (2024). Intelligent multi-objective optimization of 3D printing low-carbon concrete for multi-scenario requirements. J. Clean. Prod..

[20] Wu J., Zhao G., Wang M., Xu Y., Wang N. (2024). Concrete carbonation depth prediction model based on a gradient-boosting decision tree and different metaheuristic algorithms. Case Stud. Constr. Mater..

[21] Zhang L., Jánošík D. (2024). Enhanced short-term load forecasting with hybrid machine learning models: CatBoost and XGBoost approaches. Expert Syst. Appl..

[22] Ekanayake I.U., Meddage D.P.P., Rathnayake U. (2022). A novel approach to explain the black-box nature of machine learning in compressive strength predictions of concrete using Shapley additive explanations (SHAP). Case Stud. Constr. Mater..

[23] Bhattacharya S.K., Sahara R., Narushima T. (2020). Predicting the parabolic rate constants of high-temperature oxidation of Ti alloys using machine learning. Oxid. Met..

[24] Ye G., Wan J., Bai Y., Wang Y., Zhu B., Zhang Z., Deng Z. (2024). Prediction of the effluent chemical oxygen demand and volatile fatty acids for anaerobic treatment based on different feature selections machine-learning methods from lab-scale to pilot-scale. J. Clean. Prod..

[25] Xu H., Zou X., Sneed L.H. (2025). A two-stage classification-regression method for prediction of flexural strength of fiber reinforced polymer strengthened reinforced concrete beams. Eng. Appl. Artif. Intell..

[26] Ulloa N., León M.A.M., Palmay L.F.S., Castillo M.M. (2025). Evaluating the compressive strength of industrial wastes-based geopolymer concrete with machine learning models. Constr. Build. Mater..

[27] Hoxha E., Feng J., Sengupta A., Kirakosian D., He Y., Shang B., Gjinofci A., Xiao J. (2025). Contrastive learning for robust defect mapping in concrete slabs using impact echo. Constr. Build. Mater..

[28] Nassar A.K., Kathirvel P., Murali G., Krishna A. (2025). Development and performance evaluation of novel sustainable one-part alkali-activated fibrous concrete subjected to drop weight impact loading: An experimental study. Constr. Build. Mater..

[29] Wu Y., Zhou Y. (2022). Hybrid machine learning model and Shapley additive explanations for compressive strength of sustainable concrete. Constr. Build. Mater..

[30] Abbas Y.M., Alsaif A. (2024). Influence of feature-to-feature interactions on chloride migration in type-I cement concrete: A robust modeling approach using extra random forest. Mater. Today Commun..

